# Omega-3 EPA Supplementation Shapes the Gut Microbiota Composition and Reduces Major Histocompatibility Complex Class II in Aged Wild-Type and APP/PS1 Alzheimer’s Mice: A Pilot Experimental Study

**DOI:** 10.3390/nu17071108

**Published:** 2025-03-21

**Authors:** Barbara Altendorfer, Ariane Benedetti, Heike Mrowetz, Sabine Bernegger, Alina Bretl, Julia Preishuber-Pflügl, Diana Marisa Bessa de Sousa, Anja Maria Ladek, Andreas Koller, Pauline Le Faouder, Justine Bertrand-Michel, Andrea Trost, Ludwig Aigner

**Affiliations:** 1 Institute of Molecular Regenerative Medicine, Paracelsus Medical University, 5020 Salzburg, Austria; barbara.altendorfer@pmu.ac.at (B.A.); heike.mrowetz@pmu.ac.at (H.M.); sabine.bernegger@pmu.ac.at (S.B.); alina.bretl@pmu.ac.at (A.B.); diana.bessa@stud.pmu.ac.at (D.M.B.d.S.); 2 Institute of Experimental Neuroregeneration, Paracelsus Medical University, 5020 Salzburg, Austria; ariane.benedetti@pmu.ac.at; 3 Research Program for Experimental Ophthalmology and Glaucoma Research, Department of Ophthalmology and Optometry, University Hospital of the Paracelsus Medical University, 5020 Salzburg, Austria; j.preishuber-pfluegl@salk.at (J.P.-P.); ladek.a111@gmail.com (A.M.L.); a.koller@salk.at (A.K.); a.zurl@salk.at (A.T.); 4 MetaToul-Lipidomique Core Facility, I2MC, Inserm 1048, 31432 Toulouse, France; pauline.le-faouder@inserm.fr (P.L.F.); justine.bertrand-michel@inserm.fr (J.B.-M.); 5 Austrian Cluster of Tissue Regeneration, 1200 Vienna, Austria

**Keywords:** omega-3 polyunsaturated fatty acids, eicosapentaenoic acid (EPA), microglia, gut microbiota, MHCII, Alzheimer’s disease, APP/PS1, eicosanoids, phagocytosis, lipid droplets

## Abstract

**Background/Objectives**:**** Neuroinflammation, a hallmark of Alzheimer’s disease (AD), is characterized by elevated levels of inflammatory signaling molecules, including cytokines and eicosanoids, as well as increased microglial reactivity, and is augmented by gut microbiota dysbiosis via the gut–brain axis. We conducted a pilot experiment to elucidate the anti-inflammatory effects of dietary omega-3 polyunsaturated fatty acid (ω-3 PUFA) eicosapentaenoic acid (EPA) on the gut microbiota and neuroinflammation. **Methods**: Female APP/PS1 mice (TG) and non-transgenic littermates (WT), 13–14 months old, were fed a diet supplemented with 0.3% EPA or control chow for 3 weeks. The gut microbiota composition, hippocampal and plasma eicosanoids levels, platelet activation, and microglial phagocytosis, as well as the brain and retinal genes and protein expression, were analyzed. **Results**: EPA supplementation decreased the percentage of *Bacteroidetes* and increased bacteria of the phylum *Firmicutes* in APP/PS1 and WT mice. Inflammatory lipid mediators were elevated in the hippocampus of the TG mice, accompanied by a reduction in the endocannabinoid docosahexaenoyl ethanolamide (DHEA). Dietary EPA did not affect hippocampal lipid mediators, but reduced the levels of arachidonic-derived 5-HETE and *N*-arachidonoylethanolamine (AEA) in WT plasma. Moreover, EPA supplementation decreased major histocompatibility complex class II (MHCII) gene expression in the retina in both genotypes, and MHCII+ cells in the hippocampus of TG mice. **Conclusions**: This pilot study showed that short-term EPA supplementation shaped the gut microbiota by increasing butyrate-producing bacteria of the *Firmicutes* phylum and decreasing Gram-negative LPS-producing bacteria of the *Bacteroidetes* phylum, and downregulated the inflammatory microglial marker MHCII in two distinct regions of the central nervous system (CNS). Further investigation is needed to determine whether EPA-mediated effects on the microbiome and microglial MHCII have beneficial long-term effects on AD pathology and cognition.

## 1. Introduction

Alzheimer’s disease (AD), the most prevalent form of senile dementia, affects more than 55 million people worldwide [1]. The exact etiology of this neurodegenerative disease remains to be elucidated, but age is recognized as the predominant risk factor, with the majority of cases manifesting after the age of 65 (late-onset AD) [2]. Genetic predisposition also plays a major role, with APOE4 being the strongest risk factor for late-onset AD [3]. Inherited causal mutations in amyloid precursor protein (APP), presenilin-1 (PS1), and presenilin-2 (PS2) have been linked to early-onset familial AD, and account for a very small percentage of AD cases [4]. Notwithstanding the different onset and varying progression rates, the basal brain pathology is comparable between early-onset and late-onset AD. The neuropathological process underlying AD is complex and includes multifactorial features. The brain tissue of patients affected by AD is characterized by extracellular accumulation of amyloid-beta (Aβ) and intracellular deposits of hyperphosphorylated tau protein, as well as neuroinflammation [5]. The hallmarks of neuroinflammation include elevated levels of pro-inflammatory cytokines and chemokines, as well as lipid mediators, particularly eicosanoids derived from omega-6 arachidonic acid (e.g., leukotrienes, prostaglandins, thromboxanes, and hydroxyeicosatetraenoic acids) [6]. Microglial cells, the innate immune cells and professional phagocytes of the central nervous system (CNS), play a pivotal role in neuroinflammation and disease progression. They fail to clear aggregated protein deposits and start to produce high levels of pro-inflammatory signaling molecules, becoming neurotoxic to their environment [7].

In addition, gut microbiota dysbiosis has been associated with AD pathology, contributing to neuroinflammation and affecting microglia via the gut–brain-axis [8]. Increased abundance of the Gram-negative bacterium *Bacteroides* has been associated with more severe AD pathology [9], strengthened by the finding of reduced amyloid plaque clearance by microglia after administration of *Bacteroides* species [10]. Furthermore, fecal transplantations of AD patients into transgenic mice aggravated pathological manifestations, increased lipid mediators derived from omega-6 arachidonic acid, and influenced microglial morphology [11].

Omega-3 polyunsaturated fatty acids (ω-3 PUFAs) have been shown to exert anti-inflammatory properties in many different settings [12], and have been successful in improving cognition and alleviating symptoms of AD in animal studies [13]. In contrast, clinical trials in AD patients have been largely unsuccessful [14], and a beneficial effect of ω-3 supplementation has only been demonstrated in patients with mild cognitive impairment [15,16]. These studies focused on docosahexaenoic acid (DHA) supplements, or DHA in combination with a lower dose of eicosapentaenoic acid (EPA). Although DHA is the most abundant ω-3 PUFA in the mammalian CNS, a relatively high accumulation of EPA is found in the membranes of microglia [17]. Both ω-3 PUFAs, DHA and EPA, can be metabolized to lipid mediators with anti-inflammatory effects, in contrast to the pro-inflammatory molecules derived from arachidonic acid. EPA is metabolized more rapidly than DHA [18], competes for the same enzymes as arachidonic acid, and may therefore also inhibit the formation of pro-inflammatory ω-6 eicosanoids [19]. AD and depression both share the characteristics of neuroinflammation triggered by microglia, and clinical trials in which ω-3 PUFAs were used to treat depression were more successful when the proportion of EPA was greater than 60% of the total amount of EPA and DHA in the supplement [20].

This raises the question of whether EPA is suitable, and possibly preferable to DHA, in counteracting microglia-driven neuroinflammation in AD. Furthermore, little is known about the effects of different ω-3 PUFAs on the microbiome, and how this influences neuroinflammation in AD [21]. Before comparing the influence of EPA and DHA on AD-driven neuroinflammation, we conducted a pilot experiment to evaluate if and how high-dose EPA supplementation modulates the gut microbiota and counteracts microglial inflammation. The outcome will provide an indication of whether gut–brain axis communication needs to be considered in the follow-up project.

With this pilot study, we aimed to elucidate the potential effects of high-dose dietary EPA supplementation on the gut microbiome, microglia, neuroinflammation, and Aβ pathology in an APP/PS1 mouse model of AD. The APP/PS1 mouse model was chosen because of its well-reported features of microglial neuroinflammation [22] and gut microbiota dysbiosis [23], and our own findings of the involvement of peripheral inflammation [24,25].

After 3 weeks of EPA supplementation, we analyzed the composition of the gut microbiota and different blood parameters potentially influenced by EPA (i.e., eicosanoids, endocannabinoids, and platelets). Furthermore, two different regions of the CNS were collected for analysis: the retina, which is considered the window to the brain [26], and the hippocampus, an important cognitive structure affected by AD.

## 2. Materials and Methods

### 2.1. Animals

Female transgenic APP/PS1 (TG) mice (B6.Cg-Tg(APPswe,PSEN1dE9)85Dbo/Mmjax, Jackson Laboratory, RRID:MMRRC_034832-JAX, http://www.jax.org/strain/005864; access date 13 February 2020), 13–14 months old at the start of the experiment, and their female non-transgenic littermates, hereafter referred to as wild-type (WT) mice, were used. Mice were group-housed at the Paracelsus Medical University Salzburg under standard conditions, at a temperature of 22 °C and under a 12 h light/dark cycle, with ad libitum access to food and water. Animal care, breeding, handling, genotyping, and experiments were approved by local ethics committees (BMWFW, license numbers 2023-0.158.423 and 2021-0.009.986). The animals were bred and housed under specific pathogen-free conditions. One week before the start of the experiment, they were transferred to the conventional facility with individually ventilated cages.

### 2.2. Dietary Supplementation

EPA fish oil was extracted from commercially available ‘OMEGA 3 EPA’ capsules, kindly provided by the company Nahani (Errenteria, Spain), and mixed into standard chow (#3430, KLIBA NAFAG/Granovit, Kaiseraugst, Switzerland) to achieve a concentration of 0.3% EPA. Assuming a mouse chow consumption of 4 g per day (see Table 1) (resulting in 12 mg EPA per day), the EPA dose (461.5 mg/kg body weight; calculating with mouse body weight of 26 g) is equivalent to a human dose of 37.5 mg/kg, or approximately 2 g per day for a 60 kg person [27]. For the control diet, the same standard diet underwent the process of crushing and re-pelleting without adding any supplementation. TG and WT mice were randomly assigned cage-wise to either the control or EPA-supplemented diet for 3 weeks (*n* = 4 per group). Table 1 shows the weight of the animals before and after the experiment, the mean food consumption per day, and the increase in weight during the experiment.

### 2.3. Tissue Sampling and Processing

At the end of the experiment, mice were anesthetized with a solution of ketamine (20.5 mg/mL, Ketamidor, Richter Pharma, Wels, Austria), xylazine (5.36 mg/mL, Chanazine, Chanelle, Loughrea, Ireland), and acepromazine (0.27 mg/mL, Vanastress, Vana GmbH, Vienna, Austria) in 1× PBS, resulting in 205 mg/kg (body weight) ketamine and 53.6 mg/kg (body weight) xylazine. Blood was collected by cardiac puncture using EDTA (0.1 M)-coated syringes and BD Microlance 23G × 1” needles. To further prevent blood coagulation, EDTA (0.1 M) was added to all samples at a 1:10 ratio. A volume of 50 µL of the blood was used for general blood analysis via an automated hematology analyzer (Sysmex pocH-100iV Diff; Sysmex Europe GmbH, Vienna, Austria), and 300 µL was centrifuged for plasma collection (2000× *g*, 4 °C, 10 min; followed by an additional 15 min centrifugation after separating the liquid phase). Washed platelets were prepared from the rest of the blood for the platelet activation assay. After blood collection, animals were transcardially perfused with 20 mL of ice-cold PBS (#14287072, Gibco/Thermo Fisher Scientific, Vienna, Austria).

Collection of the eyes was performed as described previously [28]. In short, the eyes were plucked from the eye sockets, and the left eye of each mouse was fixed by immersion in 4% paraformaldehyde (PFA) for 1 h at room temperature (RT), washed in 0.1 M phosphate buffer overnight at RT, and transferred into phosphate buffer containing 15% sucrose for 24 h. The eye was frozen at −80 °C by using 2-methylbutane (GPR RECTAPURE, VWR International, Vienna, Austria), and stored at −20 °C. The retina was dissected from the right eye, snap-frozen in liquid nitrogen, and stored at −80 °C until RNA extraction.

The brain was extracted and split into its hemispheres. The left hemisphere was fixed in 4% PFA for 4 h, washed in PBS, and cryopreserved in a 30% sucrose solution. It was then cut on dry ice with a Leica microtome into 40 μm thick sagittal sections, to divide the hemisphere into 10 representative parts, as described previously [29]. Sections were stored at −20 °C in cryoprotection solution (ethylene glycol, glycerol, 0.1 M sodium phosphate buffer pH 7.4, 1:1:2 by volume) until immunohistochemical staining.

The hippocampus of the right hemisphere was extracted, snap-frozen, and stored at −80 °C until eicosanoid analysis. The rest of the right hemisphere was stored in tissue storage solution (Miltenyi, Bergisch Gladbach, Germany) on ice until microglia isolation (maximum storage time: 2 h). Feces were collected out of the rectum, snap-frozen, and stored at −80 °C until microbiome analysis.

### 2.4. Platelet Activation

The whole procedure was performed at RT and adapted from a published protocol [30]. Blood (1:10 diluted with 0.1 M EDTA) was diluted by adding 2× volume of Tyrode’s buffer (134 mM NaCl, 12 mM NaHCO_3_, 2.9 mM KCl, 1 mM MgCl_2_, 0.34 mM Na_2_HPO_4_, 10 mM Hepes solution) and centrifuged (5 min, 300× *g*). The whole layer containing platelet rich plasma (PRP) was taken and centrifuged again (12 min, 200× *g*). After this, only the upper two-thirds were collected. PRP was diluted 1:20 in flow-buffer (phosphate-buffered saline (PBS) + 2% bovine serum albumin (BSA) + 2 mM EDTA), and antibodies were added for flow cytometry analysis. The following antibodies from BD Biosciences/Becton Dickinson Austria GmbH (Vienna, Austria) were used in 1:100 dilution: CD9-PE clone KMC.8, CD62P-FITC clone RB40.34, and isotype control FITC rat IgG1 λ. After 20 min incubation in the dark, reaction was stopped via 1:6 dilution with flow-buffer, and subsequently analyzed with a BD Accuri C6 Plus Flow Cytometer (BD Biosciences/Becton Dickinson Austria GmbH, Vienna, Austria). Platelets were defined based on their forward and sideward scattering patterns on a logarithmic scale and positive staining for CD9, a marker highly expressed on platelets. CD9+ cells were gated for CD62P+ expression, which is only expressed at the platelet surface upon activation [31].

### 2.5. Microglia Isolation

An adult brain dissociation kit (Miltenyi) was used to dissociate the brain tissue, followed by magnetic bead separation (Miltenyi) of CD11b+ microglial cells. The whole procedure was performed following the protocol provided by Miltenyi [32]. In short, brain tissue was enzymatically dissociated at 37 °C on a gentleMACS Dissociator (Miltenyi) for 30 min. All the following steps were performed at 4 °C or on ice. After a debris removal and washing step, red blood cells were lysed. Following another washing, cells were solved in PBS + 0.5% BSA, and incubated 1:10 with CD11b MicroBeads (Miltenyi) for 15 min. Subsequently, cell suspension was loaded on an MS column (Miltenyi) which was placed in the magnetic field of an Octo MACS Separator (Miltentyi). Labeled cells captured in the column were eluted with PBS + 0.5% BSA. Cells were counted, and 2 × 10^4^ cells were seeded per well in 100 µL media (Advanced DMEM/F-12 with 5% fetal bovine serum, 1% pen/strep, 2.5 mM L-glutamine and 2% B27 supplement (Gibco)) in a poly-L-ornithine-coated 96-well plate, and kept at 37 °C and 5% CO_2_. Approximately 24 h after seeding, half of the media was exchanged, and on the 3rd day, a phagocytosis assay was performed.

### 2.6. Microglia Phagocytosis

Beta-Amyloid (1-42), HiLyte™ Fluor 488-labeled (0.1 mg, Anaspec, Fremont, CA, USA), henceforth referred to as Aβ-488, was solved in 50 µL NH_4_OH, diluted with PBS to a concentration of 100 µM, and stored in aliquots on −20 °C until usage. Adult primary microglia were incubated with 1 µM Aβ-488 for 4 h at 37 °C and 5% CO_2_. Subsequently, the medium was removed, and after one washing step with PBS, pre-warmed Trypsin/EDTA was added for cell detachment. After 5 min incubation at 37 °C, 1× volume of medium was added, and cells were measured on a BD Accuri C6 Plus flow cytometer (BD Biosciences). Microglia were gated by forward/sideward scatter, and the threshold for phagocytosing cells was set based on the negative control without added Aβ-488.

### 2.7. Quantification of Endocannabinoids, Endocannabinoid-like Amides, and Free Oxylipins/Eicosanoids

Endocannabinoids, including endocannabinoid-like amides, and free oxylipins were quantified in the hippocampal tissue of the right hemisphere at MetaToul-Lipidomique Core Facility [33] (I2MC, Inserm 1297, Toulouse, France), MetaboHUB-ANR-11-INBS-0010; doi.org/10.15454/VRJK-KY76. All tissues were snap-frozen with liquid nitrogen immediately after collection, and stored at −80 °C until extraction. For extraction, each frozen tissue was crushed with a FastPrep^®^-24 Instrument (MP Biomedical, Illkirch Cedex, France) in 400 µL of methanol, and 7.5 µL of endocannabinoid internal standard and 40 µL of oxylipin internal standard (Deuterium labeled compounds: LxA4-d5, LTB4-d4, 5-HETE-d8, OEA-d4) were added to homogenates of samples. After 2 crush cycles (6.5 m/s, 30 s), a volume equivalent to 0.5 mg of sample was withdrawn for protein quantification. A 260 µL volume of cold methanol was added to the supernatants. After centrifugation at 5000× *g* for 15 min at 4 °C, the supernatants were transferred into 2 mL 96-well deep plates, and diluted in H_2_O to 2 mL. Samples were then submitted to solid phase extraction (SPE) using an OASIS HLB 96-well plate (30 mg/well, Waters, Saint-Quentin-en-Yveline, France) pretreated with ethyl acetate (0.75 mL), methanol (0.75 mL), and water (0.75 mL). After sample application, extraction plate was washed with 10% MeOH (1 mL). After drying under aspiration, lipid mediators were eluted with 0.3 mL of acetonitrile, 0.4 mL of MeOH, and 0.3 mL of ethyl acetate. Before LC-MS/MS analysis, samples were evaporated under nitrogen gas and reconstituted in 15 µL of MeOH. For LC-MS/MS analyses, lipid mediators were separated on a ZorBAX SB-C18 column (2.1 mm, 50 mm, 1.8 µm) (Agilent Technologies, Santa Clara, CA, USA) using an Agilent 1290 Infinity HPLC system (Agilent Technologies) coupled to an ESI-triple quadruple G6460 mass spectrometer (Agilent Technologies). Data were acquired in Multiple Reaction Monitoring (MRM) mode with optimized conditions (ion optics and collision energy). Peak detection, integration, and quantitative analysis were performed using Mass Hunter Quantitative analysis software (version B.09.00, Agilent Technologies) based on calibration lines built with commercially available eicosanoids standards (Cayman Chemicals, Ann Arbor, MI, USA). From the target molecules (6-keto-prostaglandin (PG) F1a, resolvin (Rv) E1, thromboxane (TX) B2, 11B-PGF2a, PGE3, PGF2a, PGE2, RvD3, lipoxin (LX) B4, PGD2, RvD2, LXA4, RVD1, 8isoPGA2, leukotriene (LT) B5, PGA1, maresin 1 (MaR1), protectin Dx (PDx), RvD5, LTB4, 18-hydroxyeicosapentaenoic acid (18-HEPE), 5,6-dihydroxy-eicosatetraenoic acid (5,6-DiHETE), 15dPGJ2, 13-hydroxyoctadecadienoic acid (13-HODE), 9-HODE, 17-hydroxydocosahexaenoic acid (17-HDoHE), 14-HDoHE, 8-hydroxyeicosatetraenoic acid (HETE), 15-HETE, 12-HETE, 5-HETE, 14,15-epoxyeicosatrienoic acid (14,15-EET), 5-oxoeicosatetraenoic acid (5-oxoETE), 11,12-EET, 8,9-EET, 5,6-EET, eicosapentaenoyl ethanolamide (EPEA), palmitoylethanolamide (PEA), docosahexaenoyl ethanolamide (DHEA), *N*-arachidonoylethanolamine (AEA), 2-arachidonoylglycerol (2-AG), oleoylethanolamide (OEA), and stearoylethanolamide (SEA)), 26 could be measured in the hippocampus (6kPGF1a, TXB2, PGF2a, PGE2, PGD2, 8isoPGA2, 15dPGJ2, 13-HODE, 9-HODE, 15-HETE, 17-HDoHE, 14-HDoHE, 8-HETE, 12-HETE, 5-HETE, 14,15-EET, 5oxoETE, 11,12-EET, 8,9-EET, 5,6-EET, PEA, DHEA, AEA, 2-AG, OEA, SEA) and 13 in the blood (13-HODE, 9-HODE, 15-HETE, 14-HDoHE, 8-HETE, 12-HETE, 5-HETE, PEA, DHEA, AEA, 2-AG, OEA, and SEA).

### 2.8. Microbiome

Feces samples were sent to GVG Diagnostics GmbH (Leipzig, Germany) for microbiome analysis. DNA was extracted from feces samples using an optimized column DNA purification kit (NEBNext Microbiome DNA Enrichment kit (New England Biolabs, Ipswitch, MA, USA), according to the manufacturer’s protocol. The DNA concentration was determined by fluorometric measurement using a Qubit Fluorometer (Thermo Fisher Scientific, Waltham, MA, USA) during all preparatory steps. In addition, the integrity of the isolated DNA was checked by using a Genomic DNA ScreenTape assay (Agilent Technologies), which provided an objective assessment of the sample integrity during all steps. A microbiome DNA enrichment kit was employed to remove large amounts of host DNA. Surplus mouse DNA was extracted from the samples by selective binding and removing CpG-methylated host DNA. The high-quality DNA obtained was subsequently used for enzymatic fragmentation and library preparation, with the quantities normalized to optimal loading concentrations and sequenced (2× 150 bp) using an Illumina NextSeq (Illumina, San Diego, CA, USA). After the shotgun sequencing process, the sequence data obtained were de-multiplexed according to their nucleotide bar codes and employed for taxonomic profiling. Data analysis and taxonomic profiling was performed with CLC Genomics Workbench 20.0.03, including the Microbial Genomics module (Qiagen, Hilden, Germany) and the GVG Bioinformatic Toolbox 1.5 (GVG Diagnostics GmbH).

### 2.9. RNA Isolation of Retinal Tissue and Gene Expression Analysis

Retinal RNA isolation and gene expression analysis were performed as previously described [34]. Snap-frozen retinas were homogenized in 1 mL of TRI reagent (Sigma-Aldrich, Vienna, Austria) and centrifuged (10 min, 12,000× *g*, 4 °C). The supernatant was collected and mixed with 200 μL of chloroform. After a centrifugation step (15 min, 12,000× *g*, 4 °C), the aqueous phase containing RNA was collected and mixed with 96% isopropanol (1/2 volume). For the following steps, the High Pure RNA Isolation Kit (Roche, Vienna, Austria) was used, following the manufacturers’ instructions. cDNA was synthesized from 600 ng RNA using the iscript™cDNA synthesis Kit (Bio-Rad, Vienna, Austria). Real-time quantitative PCR (RT-qPCR) was performed in a 15 μL reaction in duplicates, each containing 24 ng of cDNA template. GoTaq Probe Mastermix (Promega, Walldorf, Germany) and the following primers were used from Integrated DNA Technologies (IDT, Leuven, Belgium) (Alox5 Mm.PT.56a.30176779, Alox5ap Mm.PT.58.5140995, Il6 Mm.PT.56a.10005566, Il10 Mm.PT.58.13531087, Tspo Mm.PT.58.43313736, Nlrp3 Mm.PT.58.13974318, Lamp2 Mm.PT.58.13168833, Trem2 Mm.PT.58.7992121) and Thermofisher (H2-Aa Mm00439211_m1, TNF Mm00443258_m1). The RT-qPCR was performed on a thermal cycler (CFX96 Real-Time System, Bio-Rad) in a two-step cycling protocol (95 °C for 15 s, 60 °C for 30 s, 39 cycles). Expression of the target genes was normalized to the mean of three reference genes (Sdha Mm01352366_m1 (Thermo Fisher Scientific, Vienna, Austria), Psmd4 Mm.PT.56.13046188 (IDT), Gusb Mm.PT.39a.22214848 (IDT)).

### 2.10. Immunohistochemistry of Retinal Tissue

PFA-fixed retinas were incubated in 10% citrate buffer in a heat steamer (Braun, Neu-Isenburg, Germany) at 65 °C for one hour for antigen retrieval. Following extensive washing in tris-buffered saline (TBS), retinas were incubated overnight in blocking medium (0.2% fish skin gelatine (Sigma-Aldrich), 0.05% Triton X-100 (Merck, Vienna, Austria) and 1% bovine serum albumin (Roth, Karlsruhe, Germany) in TBS). Incubation with primary antibodies (goat collagen IV (ColIV), 1:300, Merck #AB769; rabbit NG2, 1:600, Merck #AB5320) was performed at 4 °C for 4 days. After three washing steps, the retinas were incubated with secondary antibodies from Thermo Fisher Scientific (dilution 1:1000) (donkey anti rabbit Alexa 647, donkey anti goat Alexa 555) and DAPI in blocking medium overnight at RT. After final washing, the retinas were mounted onto glass slides and coverslipped with ProLong Gold Antifade (molecular probes). The procedure was adapted from published protocols [34].

### 2.11. Immunohistochemistry of Brain Tissue

Immunohistochemistry on free-floating slices was performed as described previously [29]. Each staining was performed on a representative tenth of the left hemisphere. After washing off the cryoprotection solution in PBS, antigen retrieval was performed in 10% citrate buffer in a heat steamer at 100 °C for 15–20 min. For staining of MHCII, the antigen retrieval step was exchanged for incubation in 50 mM glycine for 1 h. After blocking unspecific binding sites in blocking buffer (1% BSA, 0.2% fish skin gelatin, 0.1% triton X-100 in 1× PBS), sections were incubated with primary antibodies (rabbit ColIV, 1:500 dilution, Abcam (Amsterdam, Netherlands) #236640; goat PDGFRb, 1:300 dilution, R&D Systems/Biotechne (Dublin, Ireland) #AF1042; rabbit CD68, 1:500 dilution, Abcam # 125212; goat Iba1, 1:500 dilution, Abcam #ab5076; rabbit Lamp1, 1:300 dilution, Abcam #24170; rat MHCII, 1:100 dilution, Thermofisher #14532182; rabbit Iba1, 1:500 dilution, FUJIFILM Wako Chemicals Europe GmbH (Neuss, Germany) #019-19741) overnight in blocking buffer. On the following day, the sections were extensively washed and incubated with secondary antibodies from Thermofisher (1:1000 dilution) (donkey anti rat Alexa 488, donkey anti rabbit Alexa 647, donkey anti goat Alexa 568, donkey anti rabbit Alexa 488) in blocking media for 3–4 h. For Aβ plaque staining, AmyTracker 520 (Ebba Biotech, Solna, Sweden) was added in a dilution of 1:1200 with the secondary antibodies. Lipid droplets were stained with Bodipy (Thermofisher; stock solution 1 mg/mL in DMSO) 1:1000 in PBS for 15 min [35]. For lipid droplet staining, no antigen retrieval was performed, and all solutions were used without any detergent. After final washing steps, brain slices were mounted on glass slides and coverslipped with ProLong Gold Antifade mounting media (molecular probes).

### 2.12. Microscopy and Analysis

For brain sections, pictures were taken of at least three slices per mouse, containing a dorsal hippocampal region with a triangle-shaped dentate gyrus. From each hippocampus, z-stacks of two different fields of view were captured to image the whole dentate gyrus. From each retina, z-stacks from three different fields of view, each covering superficial, intermediate, and deep plexus, were taken from the medial area. All imaging was performed on a confocal laser scanning microscope (LSM700 and LSM710, Zeiss, Vienna, Austria) using a 20× objective, except for lipid droplet imaging, where a 63x objective was used. For analysis of plaque size distribution, the whole hippocampus was imaged with a VS120 Virtual-Slide-Microscope (Olympus, Hamburg, Germany) with a 20× objective.

ColIV-stained vessels (hippocampus and retina) with a diameter below 15 μm were included in the following analysis. Vessel length was measured via the software Imaris (Bitplane, Zurich, Switzerland, version 10.0.0), using the filament tracer tool to determine the total vessel length per field of view. Pericytes (PDGFRb+ cells in brain tissue and NG2+ cells in retinal tissue) were counted manually with the software ImageJ (version 1.54f) and the plugin ‘cell counter’, based on the presence of PDGFRb+ or NG2+ signal co-localizing with relatively round nuclei (DAPI) located on vessels with a diameter below 15 μm.

The Iba1+ and CD68+ immune reactive area was determined via manual threshold setting in ImageJ and analyzed via the ‘analyze particles’ function. MHCII+ cells were manually counted with ImageJ cell counter. For analysis of microglial lipid droplets, colocalization of Iba1 and Bodipy staining was performed with Imaris, and the surface tool was used for 3D modeling of the colocalization channel to obtain the volume and number of lipid droplets. Aβ-plaques and Lamp1+ dystrophic neurites were masked with the surface tool from Imaris to obtain the count and volume [29]. Plaque size distribution was performed in ImageJ with the segmentation tool ‘Robust Automatic Threshold Selection’ and ‘analyze particles’. All analyses were conducted in a blinded manner.

### 2.13. Statistics

All statistical analyses were performed using GraphPad Prism software version 9 or 10. Normal distribution of data points was verified with the Shapiro–Wilk test. Because of the small sample size, we decided to use a two-way ANOVA to detect the main genotype- and diet-specific effects. Multiple comparisons were added in the case of a significant ANOVA test, and corrected with Šídák’s multiple comparison test. A 95% confidence interval was used, and *p*-values less than 0.05 were considered significant. Graphs are presented as the mean ± standard deviation, with all data points displayed.

## 3. Results

### 3.1. EPA Supplementation Shapes the Gut Microbiota by Increasing the Firmicutes-to-Bacteroidetes Ratio

**Figure 1:**
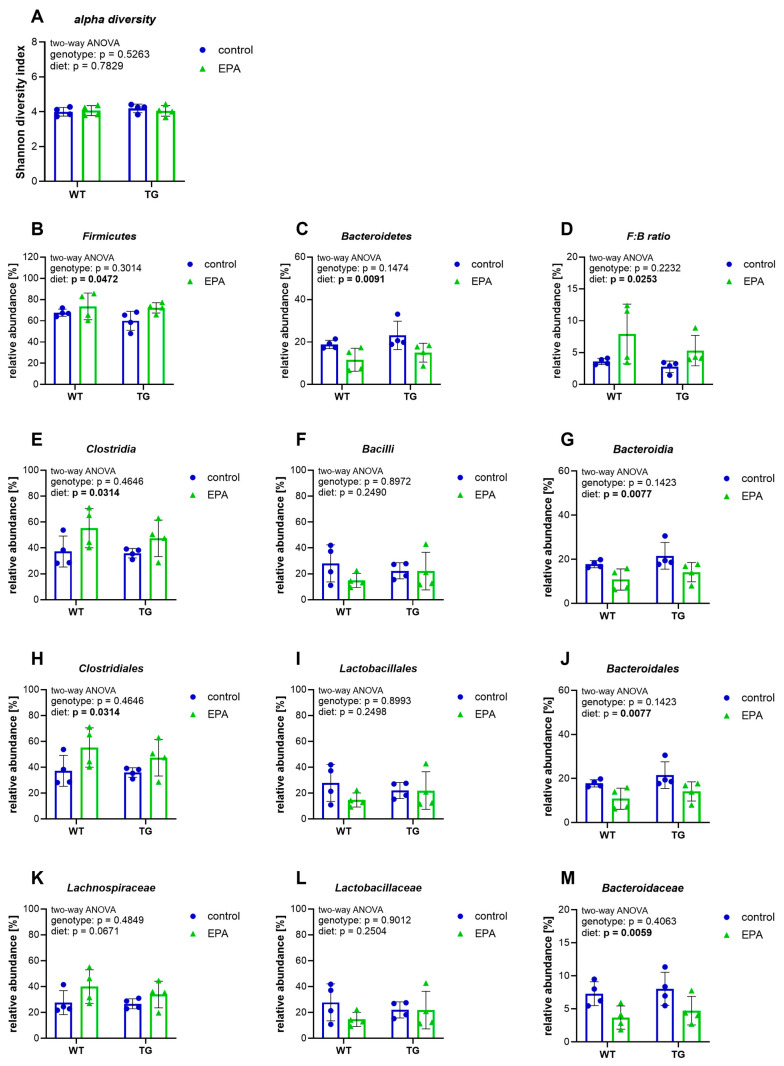
Microbiome composition. Feces were analyzed for microbiome composition; operational taxonomic units (OTUs) that were identified in all samples and exhibited an average abundance of at least 5% were included in the statistical analysis. The graphs show (**A**) the alpha diversity of the samples, indicated by the Shannon index; (**B**,**C**) the microbiota on the hierarchical taxonomic level of phylum; (**D**) the ratio of *Firmicutes* to *Bacteroidetes* bacteria; (**E**–**G**) the microbiota on the hierarchical taxonomic level of class; (**H**–**J**) the microbiota on the hierarchical taxonomic level of order; and (**K**–**M**) the microbiota on the hierarchical taxonomic level of family. (**A**–**M**) A two-way ANOVA for the main diet and genotype effects was performed, followed by Šídák’s multiple comparison test. A 95% confidence interval was used, and *p*-values less than 0.05 were considered significant. The graphs are presented as the mean ± standard deviation, with all data points shown.

Transgenic APP/PS1 (TG) mice and their non-transgenic littermates (WT) received an EPA-supplemented or control diet for three weeks to analyze the effects on several parameters of neuroinflammation. The gut microbiota are at the forefront of processing dietary intake, can adapt rapidly, and their influence on inflammatory processes in the brain is increasingly recognized. In light of this, fecal samples were analyzed for microbiome composition. On average, 334 (±46) operational taxonomic units (OTUs) were identified in each sample. In general, all samples had a high similarity, indicated by a low value of beta-diversity (0.0796). The Shannon diversity index, a measure for the diversity of the microbiota, revealed no change in the alpha diversity between treatment groups (Figure 1A). For further comparisons, we focused only on the OTUs identified in all samples and with an average abundance of at least 5%.

The most abundant bacteria phyla were *Firmicutes* (68.4% ± 8.6) and *Bacteroidetes* (17.2% ± 5.9), with no difference between WT and TG animals. EPA supplementation significantly increased the abundance of *Firmicutes* and reduced the abundance of *Bacteroidetes*, leading to a modest, but significant, increase in the ratio of *Firmicutes* to *Bacteroidetes* (F:B ratio) (Figure 1B–D). EPA supplementation effects on the gut microbiota were also observed at hierarchical lower taxonomic levels. Animals supplemented with EPA showed a higher abundance of bacteria of the class *Clostridia*, and a lower abundance of class *Bacteroidia*, while the class *Bacilli* stayed unchanged (Figure 1E–G). Bacteria of the order *Clostridiales* were elevated, while bacteria of the order *Bacteroidales* were present at lower levels with EPA supplementation (Figure 1H,J). *Lactobacillales* remained unaffected by diet (Figure 1I). At the family level, the EPA diet led to a decreased abundance of *Bacteroidaceae*, while *Lachnospiraceae* and *Lactobacillaceae* were not significantly changed (Figure 1K–M). All dietary effects on the microbiota were visible in WT and TG animals. We did not observe genotype-specific differences in the microbiome.

In short, EPA supplementation increased bacteria of the *Firmicutes* phylum, while *Bacteroidetes* were decreased, leading to an elevated *Firmicutes*:*Bacteroidetes* ratio, which is known to decline with age [36].

### 3.2. EPA Supplementation Does Not Affect Platelet Activation, but Reduces Plasma 5-HETE and AEA in Aged WT Mice

**Figure 2:**
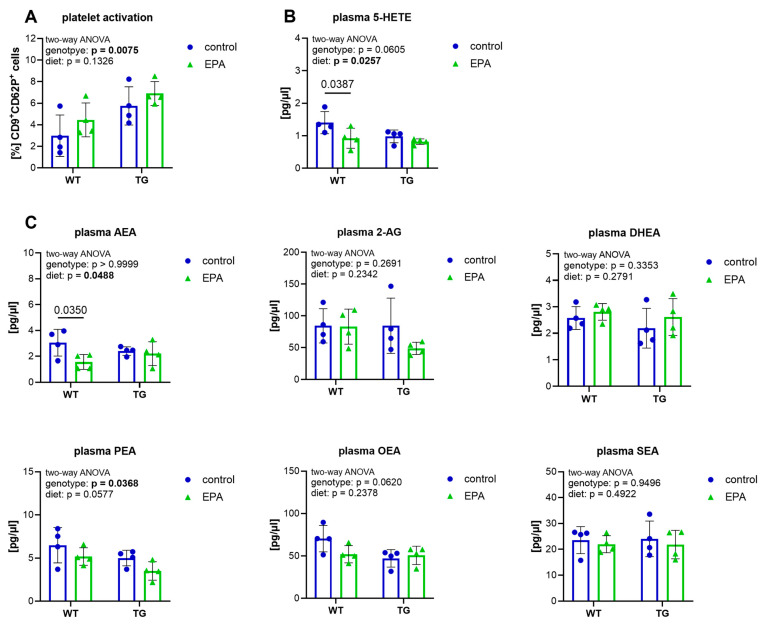
Analysis of platelet activation and plasma lipid mediators. Platelet activation was assessed via flow cytometry. CD9 was used to label platelets, and CD62P was used as an activation marker. The graphs show (**A**) the percentage of gated CD9+ platelets positive for CD62P, and the quantification of plasma (**B**) eicosanoids and (**C**) endocannabinoids and endocannabinoid-like amides. (**A**–**C**) A two-way ANOVA for the main diet and genotype effects was performed, followed by Šídák’s multiple comparison test. A 95% confidence interval was used, and p-values less than 0.05 were considered significant. The graphs are presented as the mean ± standard deviation, with all data points shown.

After being processed by the gut microbiota, nutrients are absorbed through the walls of the small intestine into the blood, before reaching other organs such as the brain. Accordingly, our next step was to analyze various blood parameters, such as blood cell counts, fatty acid-derived signaling molecules (eicosanoids, endocannabinoids and endocannabinoid-like amides), and platelet activation, to obtain an indication of the systemic inflammatory state. A general hematological analysis using an automated analyzer showed no differences between the groups (Supplemental Table S1). As ω-3 fatty acids and their eicosanoids have been suggested to play a role in platelet function [37], we measured platelet activation via the expression of the activation marker CD62P. TG mice had a higher percentage of CD62P-positive platelets than WT animals, with no significant effect of EPA supplementation (Figure 2A and Supplemental Figure S1A–F). Quantification of fatty acid-derived lipid mediators in the blood (i.e., eicosanoids, endocannabinoids and endocannabinoid-like amides) revealed no genotype-specific differences in the measured eicosanoids (5-HETE, 8-HETE, 12-HETE, 15-HETE, 9-HODE, 13-HODE, 14-HDoHE) (Figure 2B and Supplemental Figure S1G). A significant genotype effect was visible in the endocannabinoid-like amide PEA, with slightly lower levels in TG mice, and in OEA, which tended to be present at lower levels in TG animals than in WT mice (Figure 2C). AEA, 2-AG, DHEA, and SEA did not show genotype-specific effects (Figure 2C). A significant diet effect was present in 5-HETE and AEA. EPA supplementation reduced 5-HETE and AEA levels in WT mice, but not in TG animals (Figure 2B,C). PEA tended to be reduced overall with EPA supplementation, but this did not reach significance.

In summary, TG animals had higher platelet activation and lower plasma endocannabinoid-like amides than WT animals, but did not show a higher inflammatory status in the measured plasma eicosanoids. EPA supplementation decreased the pro-inflammatory eicosanoid 5-HETE and the endocannabinoid AEA in WT animals, but did not significantly affect any blood parameters measured in the TG mice.

### 3.3. Retinal Gene Expression of the Inflammatory Marker H2-Aa Is Downregulated by EPA Supplementation

**Figure 3:**
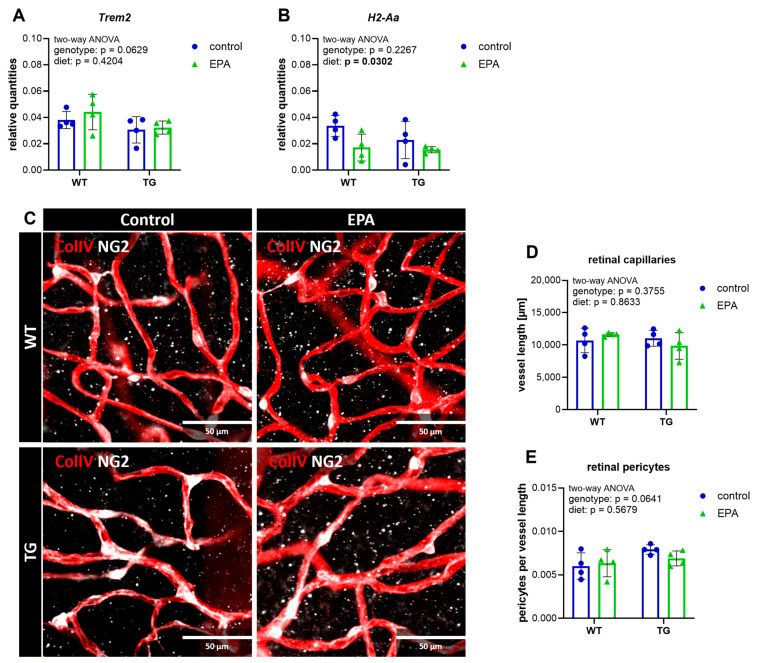
Retinal gene expression and vascular integrity. Retinal gene expression of *Trem2* (**A**) and *H2-Aa* (**B**) was analyzed with RT-qPCR. (**C**) Representative retinal tissue immunohistochemically stained with Collagen IV (ColIV) for blood vessels (in red) and neural/glial antigen 2 (NG2) for pericytes (in white); the scale size is 50 µm. (**D**) The total length of retinal capillaries per field of view. (**E**) The pericyte count per vessel length. (**A**,**B,D**,**E**) A two-way ANOVA for main diet and genotype effects was performed. A 95% confidence interval was used, and *p*-values less than 0.05 were considered significant. The graphs are presented as the mean ± standard deviation, with all data points shown.

The primary objective of this study was to investigate the effect of EPA supplementation on neuroinflammation within the CNS. To this end, two distinct regions were analyzed: the hippocampus, a central structure involved in learning and memory functions, and the retina, which can be considered the window to the brain. The retina has been identified as a site of pathological manifestation in AD, and may serve as an indicator of brain function [26]. We performed retinal gene expression analysis of several inflammatory and AD-associated markers (*Alox5*, *Alox5ap*, *H2-Aa*, *Il6*, *Il10*, *Tnf*, *Tspo*, *Nlrp3*, *Trem2*, *Lamp2*) to identify potential targets for further investigation in brain tissue. However, the retinal gene expression levels of *Alox5*, *Il6*, *Il10*, *Tnf*, *Tspo*, and *Nlrp3* were found to be too low to provide reliable results. *Alox5ap* and *Lamp2* expression did not differ between genotypes (Supplemental Figure S2A,B), and *Trem2* expression showed a tendency to be downregulated in TG animals, although this did not reach statistical significance (Figure 3A). Neither *Alox5ap*, *Lamp2*, nor *Trem2* were affected by EPA supplementation. Interestingly, retinal *H2-Aa*, the gene encoding major histocompatibility complex class II (MHCII), was significantly reduced by the EPA-supplemented diet, although it was not differentially regulated between genotypes (Figure 3B).

Deteriorations of the neurovascular unit are strongly associated with AD and neuroinflammation [38,39]. Since the APP/PS1 mouse model features substantial retinal vascular deficiencies [40], and ω-3 fatty acids are supposed to act beneficially on vascular integrity [41], we stained for blood vessels and pericytes. In contrast to published data, total capillary vessel length and pericyte density were not decreased in the TG animals, and EPA supplementation had no effect on the parameters analyzed (Figure 3C–E).

Similarly, we observed no genotype- or diet-specific effects on capillary length or pericyte density in the hippocampus (Supplemental Figure S3).

### 3.4. Higher Inflammatory Eicosanoids and Lower Endocannabinoids in the Hippocampus of APP/PS1 Mice

**Figure 4:**
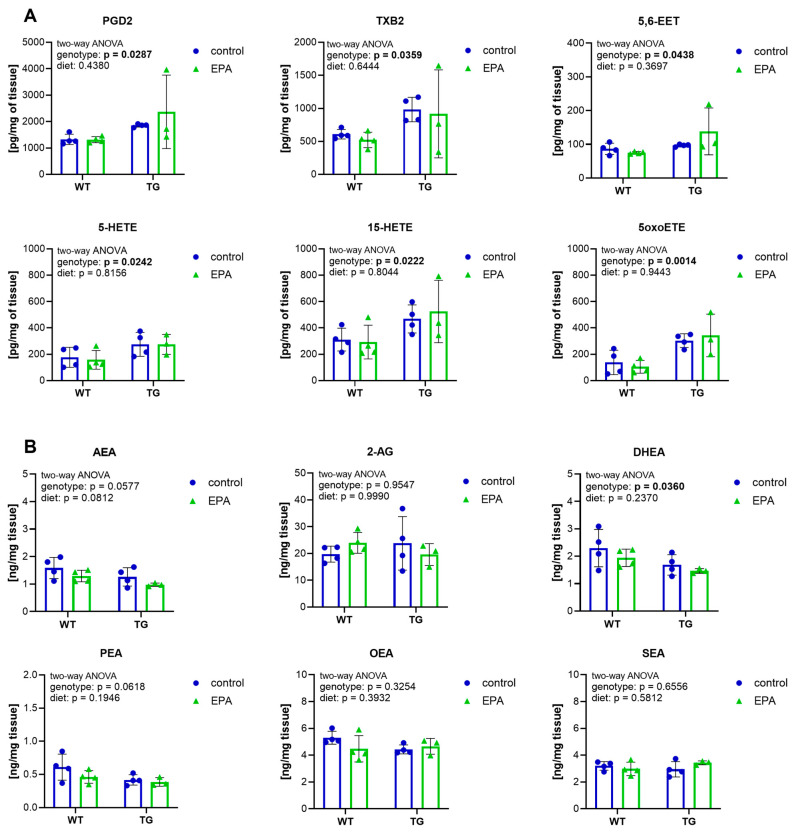
Hippocampal lipid mediators. Quantification of hippocampal (**A**) eicosanoids and (**B**) endocannabinoids and endocannabinoid-like amides. In the TG+EPA group, one of the samples was unfortunately lost during sample preparation for LC-MS/MS analysis; therefore only 3 data points are available. A two-way ANOVA for the main diet and genotype effects was performed. A 95% confidence interval was used, and *p*-values less than 0.05 were considered significant. The graphs are presented as the mean ± standard deviation, with all data points shown.

Lipid mediators play an essential role in neuroinflammation during AD progression [6]. Therefore, we measured fatty acid-derived eicosanoids, endocannabinoids, and endocannabinoid-like amides in hippocampal tissue to determine the inflammatory state in the brain of the TG AD mouse model used in this study. Among the eicosanoids quantified in the hippocampus, the arachidonic acid-derived mediators PGD2, TXB2, 5,6-EET, 5-HETE, 15-HETE, and 5oxoETE were found to be genotype-specifically increased in TG mice (Figure 4A), whereas others (6kPGF1a, PDF2a, PGE2, 8isoPGA2, 15dPGJ2, 13-HODE, 9-HODE, 17-HDoHE, 14-HDoHE, 8-HETE, 12-HETE, 14,15-EET) did not differ between genotypes (Supplemental Figure S4). EPA supplementation did not affect any of them. Among the endocannabinoids and endocannabinoid-like amides measured, AEA and PEA showed only a tendency to have lower levels in TG mice, whereas DHEA was significantly decreased in TG animals (Figure 4B). We did not observe a genotype-specific effect in the levels of 2-AG, OEA, and SEA, and EPA supplementation did not affect any of the endocannabinoids or endocannabinoid-like amides measured.

In summary, TG animals showed increased levels of inflammatory eicosanoids in the hippocampus, and reduced levels of the anti-inflammatory endocannabinoid DHEA. EPA supplementation did not affect the levels of the lipid mediators analyzed in the hippocampus.

### 3.5. EPA Reduces Hippocampal MHCII+ Cells, but Does Not Influence Microglial Phagocytosis, Aβ-Pathology, or Lipid Droplet Formation

**Figure 5:**
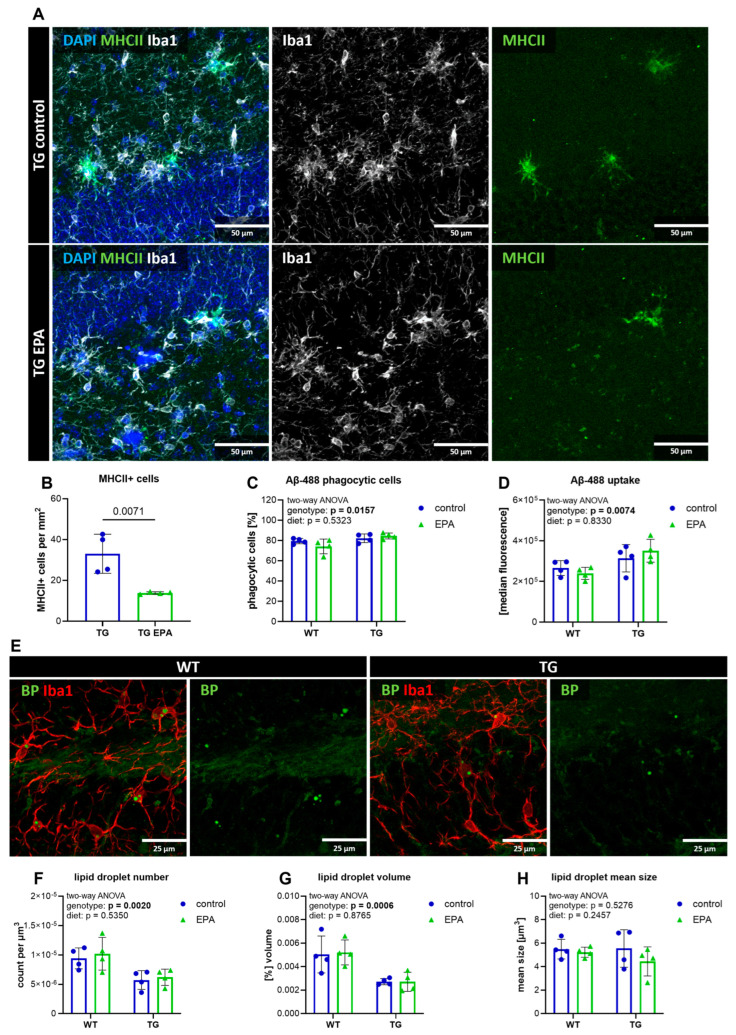
Microglia analysis. (**A**) Representative images of hippocampal brain sections stained with 4′,6-diamidino-2-phenylindole (DAPI) for cell nuclei (in blue), calcium-binding adapter molecule 1 (Iba1) for microglia (in white), and major histocompatibility complex class II (MHCII) (in green); the scale size is 50 µm. (**B**) Quantification of MHCII+ cells in TG mice. Student’s t-test was performed with a 95% confidence interval, and a p-value below 0.05 was considered significant. (**C**,**D**) Primary microglia of one hemisphere were isolated, and a phagocytosis assay with fluorescent labeled amyloid peptide (Aβ-488) was performed and measured on a flow cytometer. The graphs show the percentage of phagocytic cells (**C**) and the median fluorescence as a measure of peptide uptake (**D**). (**E**) Representative pictures of hippocampal sections stained with Bodipy for lipid droplets (in green) and Iba1 for microglia (in red); the scale size is 25 µm. The lipid droplet count (**F**), total volume per field of view (**G**), and mean volume (**H**) were measured with Imaris (Bitplane) software. (**C**,**D**,**F**–**H**) A two-way ANOVA for the main diet and genotype effects was performed. A 95% confidence interval was used, and *p*-values less than 0.05 were considered significant. The graphs are presented as the mean ± standard deviation, with all data points shown.

As the quantification of eicosanoid levels revealed a pro-inflammatory state in the hippocampus of TG animals, we analyzed whether dietary EPA influenced microglial function. Since gene expression analysis of the inflammatory marker MHCII in the retina was significantly reduced upon EPA supplementation, this prompted us to investigate whether a similar effect could be observed in brain tissue. MHCII+ cells were detected in the hippocampal tissue of the TG mice, whereas no signal was found in the WT animals. In the TG mice, EPA supplementation significantly reduced the number of hippocampal MHCII+ cells, which mainly co-localized with Iba1 staining (Figure 5A,B). Based on this encouraging downregulatory effect on a marker of pro-inflammatory microglia, we further investigated microglial phenotypic characteristics, including phagocytosis, and amyloid plaque pathology.

Ex vivo microglial phagocytosis of fluorescence-labeled Aβ-488 peptide showed a genotype-specific effect, with a higher amount of phagocytosing cells, as well as a higher uptake of Aβ-488, in the TG animals (Figure 5C,D and Supplemental Figure S5A–F). In addition, lysosomal CD68, a marker often used for phagocytic microglia, was present at much higher levels in TG animals than in WT animals (Supplemental Figure S5G,I,J), which was expected because of the Aβ pathology in TG mice. The hippocampal area occupied by Iba1+ microglia did not differ between genotypes (Supplemental Figure S5G,H). Dietary EPA did not affect ex vivo microglial phagocytosis, CD68+, or Iba1+ area (Figure 5C,D and Supplemental Figure S5). Subsequent analysis of Aβ plaque pathology in the TG animals did not show any differences in total Aβ plaque volume, count, or plaque size distribution with dietary treatment (Supplemental Figure S6A–D). In addition, the amount of dystrophic neurites around the Aβ plaques was not affected by EPA supplementation (Supplemental Figure S6A,E).

The accumulation of lipid droplets, particularly in microglial cells, is considered an age-related process. The role of lipid droplets in neurodegenerative diseases such as AD is still under investigation. To determine whether EPA supplementation affects microglial lipid droplet formation, we stained lipid droplets and Iba1+ microglia, and quantified the co-localization volume. Surprisingly, the number and volume of microglial lipid droplets were lower in TG animals (Figure 5E–G). The mean lipid droplet size did not change between genotypes (Figure 5H). Dietary EPA supplementation had no observable effect on lipid droplet accumulation (Figure 5E–H).

Overall, microglia from TG mice displayed distinct phenotypic characteristics, with increased phagocytic activity and lysosomal CD68, in addition to remarkable MHCII expression compared to WT animals. In contrast, microglial lipid droplet formation was higher in WT mice. EPA supplementation reduced MHCII+ cells, but did not affect microglial phagocytosis, lipid droplet accumulation, or Aβ pathology.

## 4. Discussion

In this pilot study, female 13–14-month-old APP/PS1 TG and WT control mice received a diet supplemented with the ω-3 PUFA EPA or a control diet for three weeks. Genotype-specific and dietary effects on several parameters of AD-related neuroinflammation were assessed in two different regions of the CNS, the retina, and the hippocampus, as well as in the blood and the gut microbiota. We demonstrated that EPA supplementation was able to modulate the composition of the gut microbiota by increasing the *Firmicutes*-to-*Bacteroidetes* ratio, reducing retinal *H2-Aa* gene expression, as well as MHCII+ cells in the hippocampus, indicating an anti-inflammatory effect on the microbiome and microglial cells. However, EPA supplementation did not alter Aβ plaque pathology, nor did we observe any other genotype-specific alterations in the AD mouse model used, such as higher platelet activation, elevated levels of eicosanoids, lower endocannabinoid DHEA levels, diminished lipid droplet accumulation, and increased microglial phagocytosis. Nevertheless, EPA supplementation did result in a decrease in the arachidonic acid-derived eicosanoid 5-HETE and the endocannabinoid AEA in WT plasma. The previously observed impairment of the neurovascular unit by AD-related inflammation [40] was not detected in this study in either retinal or hippocampal tissue.

The APP/PS1 mouse model we used in this study is widely used and well characterized. However, peripheral inflammation is hardly addressed. We recently showed an activated state in platelets in this mouse model [24], which we could also confirm here, in comparison to WT controls. Although the role of lipid mediators in neuroinflammation is increasingly recognized [6], the eicosanoid and endocannabinoid profile in the TG mice used here is not well described. The endocannabinoid system has been identified as a potential target in neurodegenerative diseases [42], but little is known about the role of different endocannabinoids in AD progression. To our knowledge, we have shown for the first time that the endocannabinoid DHEA was decreased in the hippocampus of an AD mouse model, emphasizing the role of the endocannabinoid system in AD. While no genotype-specific significant differences were observed in the plasma lipid mediator profile, elevation of pro-inflammatory eicosanoids was detected in the hippocampus. This finding is in accordance with other reports that have also found increased 5-HETE, TXB2, and PGD2 in the brain in AD mouse models [43,44,45], and elevated 15-HETE in postmortem AD brains [46].

Although the EPA-rich diet did not affect hippocampal lipid mediators, we found a dietary effect on arachidonic acid-derived inflammatory signaling molecule 5-HETE plasma levels. This is consistent with previous findings after ω-3 PUFA supplementation [47], but, so far, there is no explanation as to why we see this effect in WT and not in TG animals. Similarly, endocannabinoid AEA was reduced in the plasma of WT mice by EPA supplementation. A reduction in arachidonic acid-derived endocannabinoids caused by DHA and EPA supplementation has been shown previously [48,49], but the decrease in arachidonic acid-derived endocannabinoids was accompanied by an elevation of ω-3 PUFA-derived endocannabinoids, such as DHEA [49]. As we did not observe such a bidirectional mechanism, it may be that the supplementation has to be prolonged to induce such a compensatory mode. Otherwise, a reduction in AEA should be viewed with caution, since lower levels of AEA or increased degradation of AEA have been associated with enhanced AD pathology [50,51]. However, AEA was decreased by the EPA diet only in the plasma of WT mice, and not in the plasma of TG mice.

The accumulation of microglial lipid droplets has been associated with inflammation and microglial dysfunction in the aged brain [35], suggesting a similar role in neurodegenerative diseases such as AD. Indeed, an increase in lipid droplet-containing microglia with a similar transcriptional profile to mouse lipid droplet-accumulating microglia (LDAM) was found in the brains of AD patients recently [52]. Consequently, our observations of reduced microglial lipid droplet abundance in the TG mouse model compared to the WT mice appear contradictory. Since microglial lipid droplet formation in AD is linked to apolipoprotein E (ApoE) [52,53], and mouse ApoE differs from human ApoE [54], this could be a reason for the different results. To our knowledge, lipid droplets have been detected in the APP/PS1 mouse model [55], but have never been compared to WT controls. However, glial droplets also appear to have neuroprotective effects [56], at least in animal models. During oxidative stress, neurons transport peroxidized lipids to glial cells as a protective measure, while disruption of this transport can lead to increased neurotoxicity [53]. Glial lipid droplet formation has also been associated with neuroprotection in inflammatory conditions following ischemic stroke [57]. Therefore, a reduction in microglial lipid droplets may be an indicator of the increased oxidative stress described in AD [58] and the inability of the microglia to compensate and protect the neurons, which needs further investigation.

The role of the gut microbiota in AD, and its communication with brain cells via the gut–brain axis, is being acknowledged more and more [59]. In general, bacterial diversity is reduced in AD, and certain strains are more abundant, while others are less abundant. Studies on the microbiome are heterogeneous and highly dependent on geographical location [60]. However, most studies suggest that *Firmicutes*, *Clostridiaceae*, and *Lachnospiraceae* are decreased in AD [61,62], while *Bacteroidetes* tend to increase with age and cognitive decline [62,63]. The *Firmicutes*-to-*Bacteroidetes* (*F*:*B*) ratio is often used as an indicator of the health status of the microbiome. While a high *F*:*B* ratio is associated with obesity [64], a lower *F*:*B* ratio is found in inflammatory bowel disease [65] and in aged individuals [36]. This decrease in the *F*:*B* ratio with age was also found in APP/PS1 mice and their WT controls [66]. In our study, EPA supplementation significantly increased the butyrate-producing bacteria *Firmicutes* (at the phylum rank of classification), *Clostridia* (at the class rank of classification), and *Clostridiales* (at the order rank of classification), while Gram-negative, and therefore lipopolysaccharide-producing, bacteria were decreased at the phylum (*Bacteroidetes*), class (*Bacteroidia*), order (*Bacteroidales*), and family (*Bacteroidaceae*) levels. Available data on the effects of ω-3 supplementation on the gut microbiota in AD are relatively sparse [67]. However, in healthy middle-aged individuals, ω-3 supplementation led to a higher abundance of *Clostridiaceae* [68] and *Firmicutes*, and a reduced abundance of *Bacteroidetes* [69]. Also in animal studies, ω-3 supplementation was associated with a decrease in *Bacteroidetes* [21]. Overall, ω-3 supplementation is related to an elevation in beneficial butyrate-producing bacteria, and a reduction in more pro-inflammatory microbiota [21]. We now can confirm this change to a more anti-inflammatory and neuroprotective state of the microbiome with EPA supplementation in TG AD mice and their WT littermates. This finding is consistent with our finding of downregulation of the inflammatory marker MHCII at the gene level in the retina and at the protein level in the brains of TG mice.

MHCII is a widely used marker of microglial reactivity in AD in both animal and human studies [70,71]. It is elevated in the brains of AD patients compared to healthy controls [71], which is consistent with the increased appearance of MHCII+ cells in the AD mouse model used here. Individuals with substantial cerebral Aβ pathology, but no cognitive impairment, exhibited significantly lower levels of MHCII+ cells (and neurofibrillary tangles), in comparison to AD patients with dementia symptoms and comparable Aβ pathology [72]. In animal studies, upregulation of microglial MHCII expression was associated with enhanced neurodegeneration and memory decline in a mouse model of tauopathy [73], but also with higher phagocytosis and more efficient clearance of Aβ-plaques [74,75]. It appears to be highly context-dependent as to whether microglial MHCII upregulation is beneficial or detrimental. We have shown that short-term EPA supplementation reduced MHCII+ cells in TG mice, while microglial phagocytosis and Aβ pathology were unaltered by treatment. Whether microglial MHCII reduction may lead to improved pathology and cognition in the long term remains to be investigated.

The published finding of deficiency of retinal vascular integrity in TG mice [40] was not observed in the present study. This discrepancy may be due to differences in the age of the animals, as the present study utilized mice that were approximately three months older. It is possible that the aging effects in the WT mice masked the differences between the genotypes. Nevertheless, caution is warranted when using this mouse model for vascular studies.

### Limitations

The present study was designed as a pilot experiment to investigate the potential effects of EPA supplementation on neuroinflammation and gut microbiota, and to gain insight into the analysis that will be the focus of a future project. Therefore, the sample size was intentionally limited to minimize cost and effort, but we recognize that the study may be statistically underpowered. Although the positive effects of ω-3 fatty acids were found to be more beneficial with longer supplementation [13], we chose a shorter time period to investigate whether the effects could be observed in the brain before major changes in the microbiome occurred. The results showed that the gut microbiota had a rapid and substantial response, while the effects on the brain were modest. Therefore, we recommend that future studies of ω-3 supplementation take into account communication via the gut–brain axis. To reduce the number of animals used, only female subjects were included in this study, because females (mice and humans) are more affected by AD pathology [76,77], and thus have greater relevance. Whether an EPA diet has the same effects in male animals remains to be investigated.

## 5. Conclusions

In this pilot study, short-term dietary supplementation with ω-3 fatty acid EPA led to a beneficial shift in the composition of the gut microbiota and decreased expression of the microglial inflammatory marker MHCII. This indicates an anti-inflammatory effect of EPA in the brain and gut of a mouse model of AD, and opens the door to prolonged trials using EPA-dominated supplements with a larger sample size to investigate potential effects on cognitive performance.

## Figures and Tables

**Table 1:** Animal weight and food consumption; values in brackets: standard deviation (SD).

Group (*n* = 4)	Weight at Start [g]	Weight at End [g]	Mean Food Consumption/Day [g]	Increase in Weight [g]	Increase in Weight [%]
WT	26.4 (1.1)	29.4 (2.5)	4.9 (0.8)	3.1 (1.5)	11.4 (5.5)
WT + EPA	26.9 (4.9)	30.2 (6.5)	4.4 (0.5)	3.3 (1.7)	11.7 (5.1)
TG	25.8 (0.4)	27.4 (1.0)	4.1 (0.4)	1.6 (0.9)	6.2 (3.6)
TG + EPA	25.1 (2.4)	26.3 (2.5)	4.3 (0.5)	1.2 (0.2)	5.0 (0.5)

## Data Availability

The original contributions presented in the study are included in the article and Supplementary Materials, further inquiries can be directed to the corresponding authors.

## Supplementary Materials

The following supporting information can be downloaded at https://www.mdpi.com/article/10.3390/nu17071108/s1: Supplemental Table S1; Supplemental Figure S1—platelet activation and plasma lipid mediators; Supplemental Figure S2—retinal gene expression; Supplemental Figure S3—hippocampal vascular integrity; Supplemental Figure S4—hippocampal lipid mediators; Supplemental Figure S5—microglial phagocytosis; Supplemental Figure S6—Aβ-plaque pathology.

## Abbreviations

The following abbreviations are used in this manuscript:

2-AG	2-arachidonoylglycerol
AD	Alzheimer’s disease
AEA	*N*-arachidonoylethanolamine
ANOVA	analysis of variance
Aβ	amyloid-beta
Aβ-488	Beta-Amyloid (1-42), HiLyte™ Fluor 488-labeled
BSA	bovine serum albumin
CD	cluster of differentiation
CNS	central nervous system
ColIV	collagen IV
DAPI	4′,6-diamidino-2-phenylindole
DHA	docosahexaenoic acid
DHEA	docosahexaenoyl ethanolamide
DiHETE	dihydroxy-eicosatetraenoic acid
EDTA	ethylenediamine tetraacetic acid
EET	epoxyeicosatrienoic acid
EPA	eicosapentaenoic acid
EPEA	eicosapentaenoyl ethanolamide
HDoHE	hydroxydocosahexaenoic acid
HEPE	hydroxyeicosapentaenoic acid
HETE	eicosatetraenoic acid
HODE	hydroxyoctadecadienoic acid
Iba1	ionized calcium-binding adaptor molecule 1
Lamp1	lysosomal-associated membrane protein 1
LPS	lipopolysaccharide
LSM	laser scanning microscope
LT	leukotriene
LX	lipoxin
MaR	maresin
MHCII	major histocompatibility complex class II
NG2	neural/glial antigen 2
OEA	oleoylethanolamide
OTU	operational taxonomic unit
oxoETE	oxoeicosatetraenoic acid
P	protectin
PBS	phosphate-buffered saline
PDGFRb	platelet-derived growth factor receptor beta
PEA	palmitoylethanolamide
PFA	paraformaldehyde
PG	prostaglandin
PRP	platelet rich plasma
PUFAs	polyunsaturated fatty acids
RT	room temperature
RT-qPCR	real-time quantitative PCR
Rv	resolvin
SEA	stearoylethanolamide
TG	transgenic
TX	thromboxane
WT	wild-type
ω-3	omega-3
