# Supplementary material for: Omega-3 EPA Supplementation Shapes the Gut Microbiota Composition and Reduces Major Histocompatibility Complex Class II in Aged Wild-Type and APP/PS1 Alzheimer’s Mice: A Pilot Experimental Study
Source: Nutrients. 2025 Mar 21;17(7):1108. doi: 10.3390/nu17071108 (PMC11990804; doi:10.3390/nu17071108)
Supplement: Supplementary file 1 [file nutrients-17-01108-s001.zip › nutrients-3530955-supplementary.pdf]

Table S1

	WBC [x10^3/µL]	RBC [x10^6/µL]	HGB [g/dL]	HCT [%]	MCV [fL]	MCH [pg]	MCHC [g/dL]	PLT [x10^3/µL]
**WT**	1.58 (0.19)	7.09 (0.21)	10.05 (0.37)	30.50 (1.16)	43.03 (0.90)	14.15 (0.31)	32.95 (0.13)	985.50 (88.47)
**WT+EPA**	1.80 (1.41)	7.10 (0.39)	9.85 (0.60)	29.90 (1.62)	42.15 (0.24)	13.90 (0.29)	32.93 (0.51)	905.00 (154.82)
**TG**	1.45 (0.33)	7.14 (0.43)	9.93 (0.70)	30.08 (1.80)	42.13 (0.60)	13.90 (0.35)	32.98 (0.39)	861.75 (183.82)
**TG+EPA**	2.08 (0.73)	7.19 (0.38)	10.02 (0.54)	30.22 (1.67)	42.04 (0.82)	13.92 (0.33)	33.14 (0.13)	835.60 (165.67)

Table S1. General blood analysis via an automated hematology analyzer (Sysmex pocH-100iV Diff; Sysmex Europe GmbH). Abbreviations: WBC – white blood cells; RBC – red blood cells; HGB – hemoglobin; HCT - hematocrit; MCV - mean corpuscular volume; MCH - mean corpuscular hemoglobin; MCHC - mean corpuscular hemoglobin concentration; PLT – platelets

Figure S1 - platelet activation and plasma lipid mediators

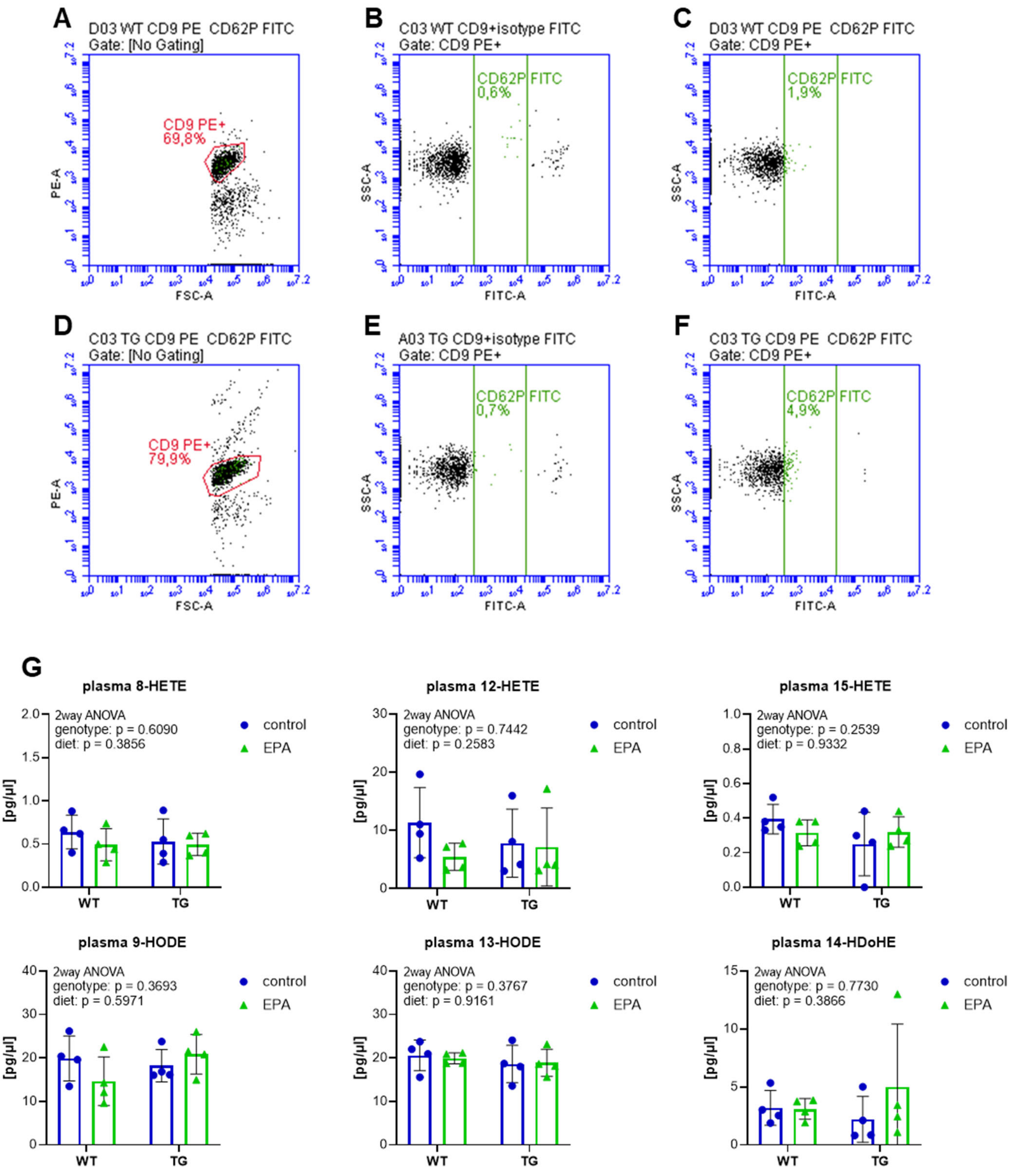

Figure S1 – platelet activation and plasma lipid mediators; (A-F) Platelets activation was assessed via flow cytometry in WT (A-C) and TG (D-F) mice. CD9 was used to gate for platelets (A+D) and a threshold for platelet activation, based on the FITC isotype (B+E) was set for the activation marker CD62P (C+F). (G) Quantification of plasma eicosanoids. Two-way ANOVA for main diet and genotype effects was performed. A 95% confidence interval was used and p-values less than 0.05 are considered significant. Graphs are presented as mean ± standard deviation with all data points shown.

Figure S2 – retinal gene expression

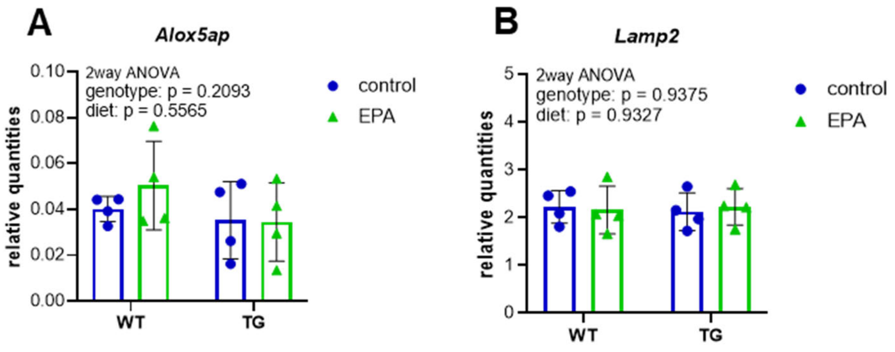

Figure S2 – retinal gene expression; Retinal gene expression of Alox5ap (**A**) and Lamp2 (**B**) was analyzed with RT-qPCR. Graphs show gene expression relative to housekeeping genes. Two-way ANOVA for main diet and genotype effects was performed. A 95% confidence interval was used and p-values less than 0.05 are considered significant. Graphs are presented as mean ± standard deviation with all data points shown.

Figure S3 – hippocampal vascular integrity

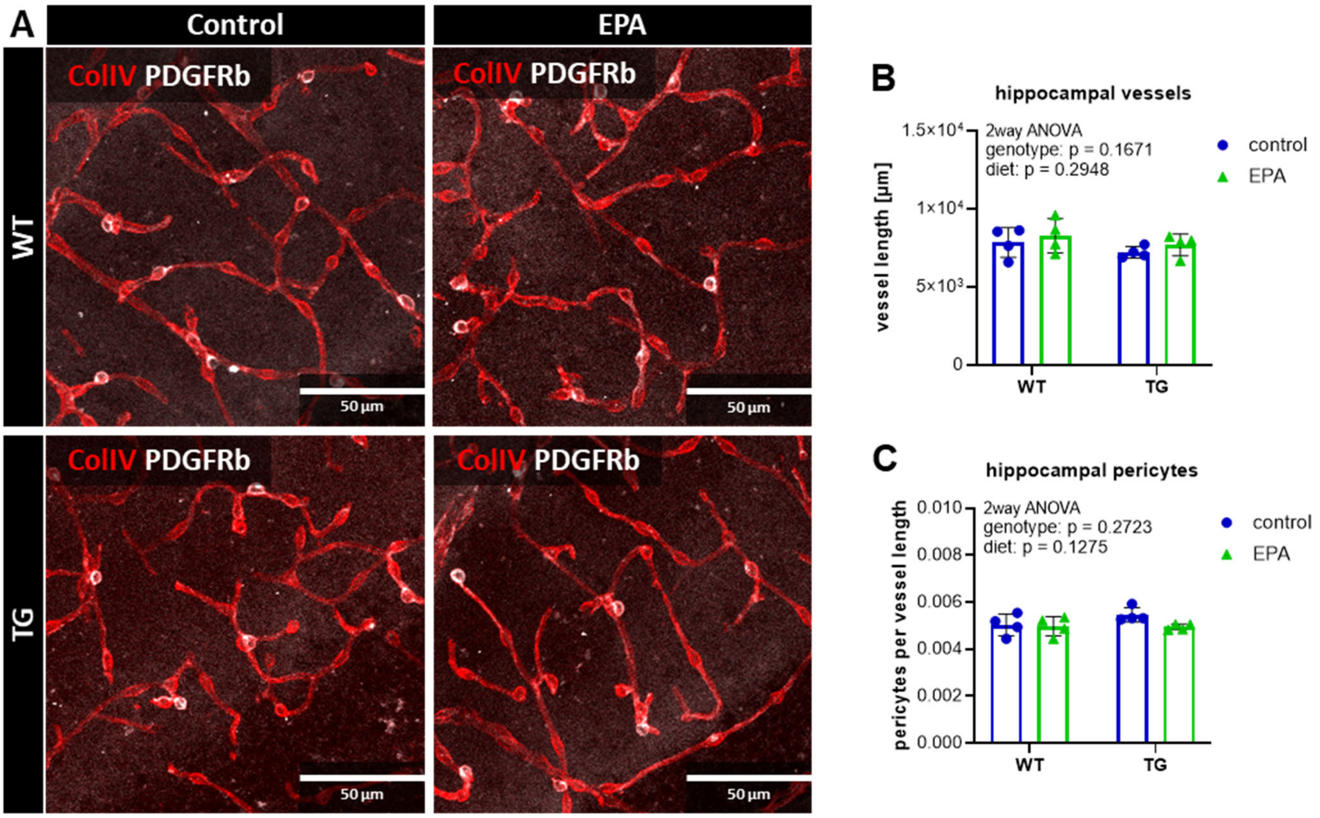

Figure S3 – hippocampal vascular integrity; (A) Representative hippocampal sections immunohistochemically stained with Collagen IV (ColIV) for blood vessels (in red) and platelet-derived growth factor receptor beta (PDGFRb) for pericytes (in white), scale size is 50 µm. (B) Total length of retinal capillaries per field of view. (C) Pericyte count per vessel length. (B+C) Two-way ANOVA for main diet and genotype effects was performed with a 95% confidence interval. P-values less than 0.05 are considered significant. Graphs are presented as mean ± standard deviation with all data points shown.

Figure S4 - hippocampal lipid mediators

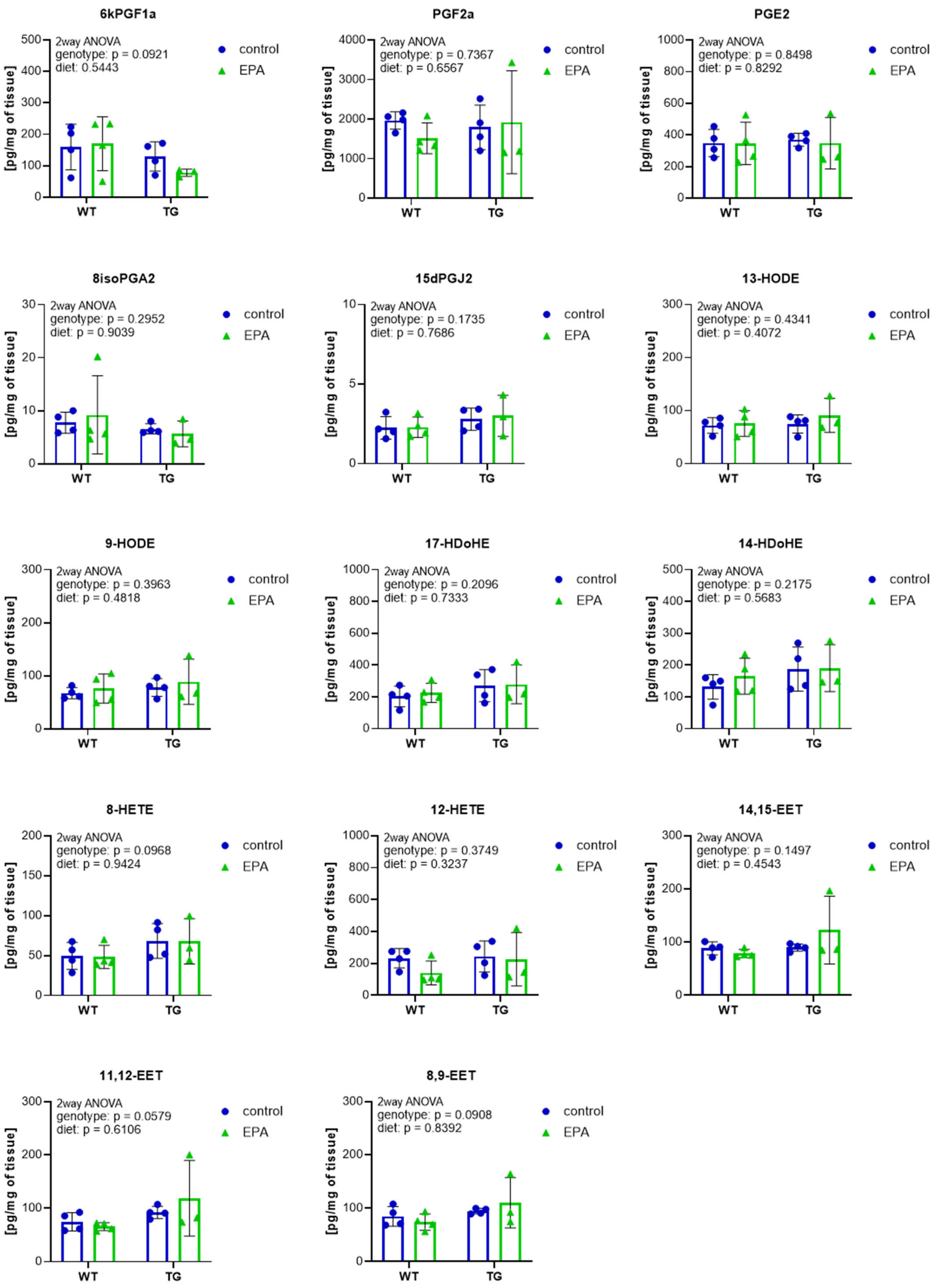

Figure S5 – microglial phagocytosis

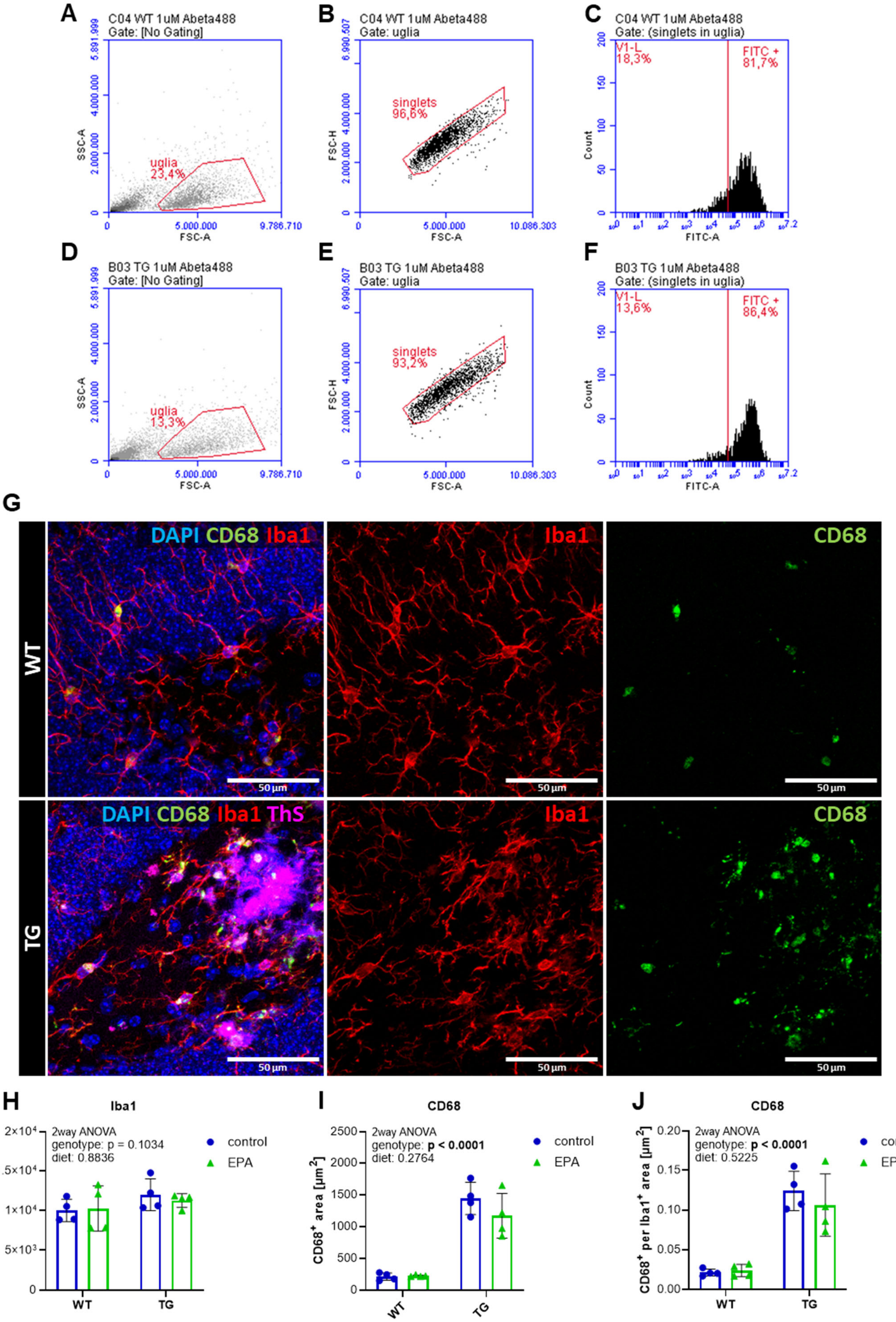

Figure S6 **-** Aβ-plaque pathology

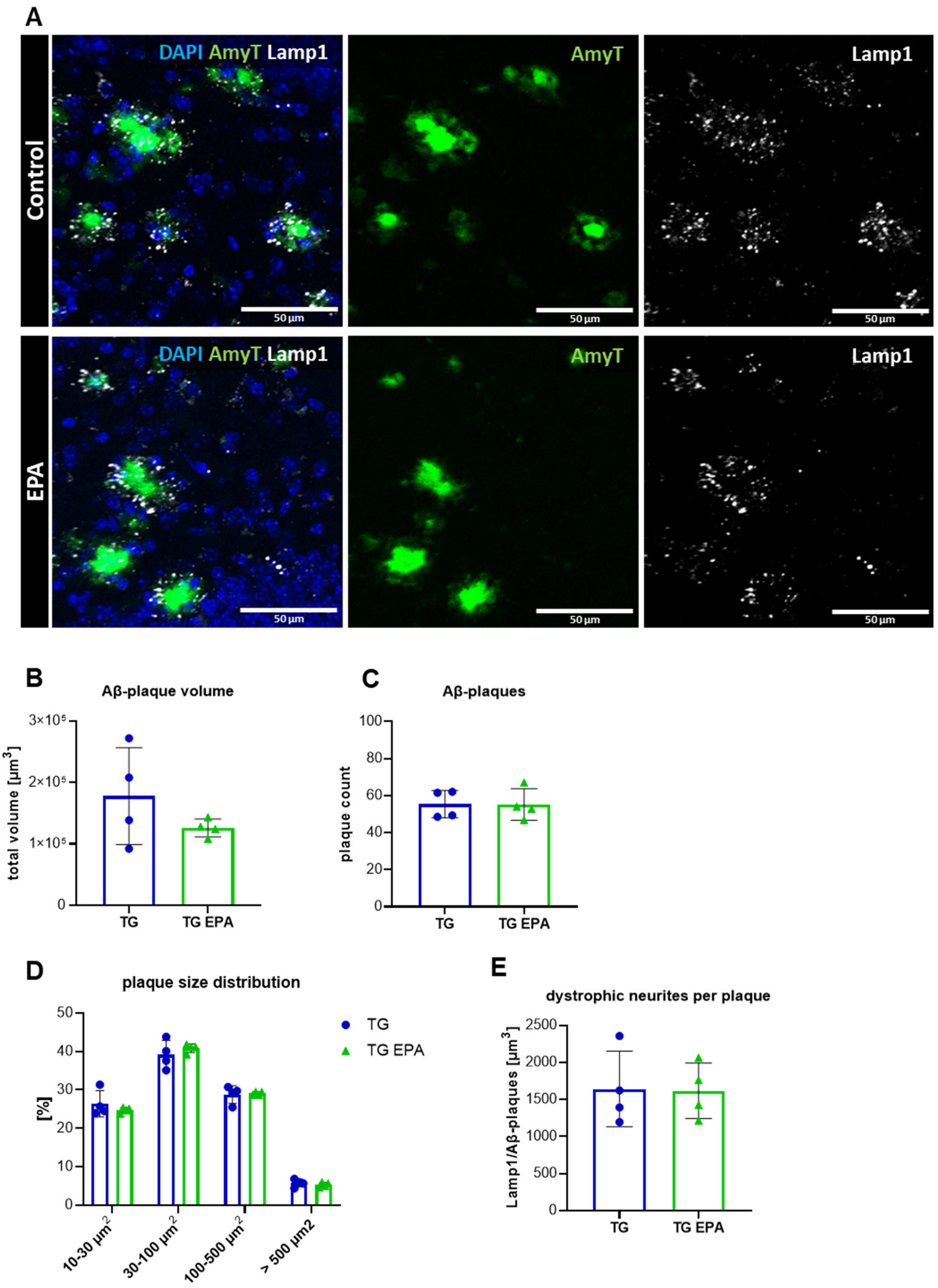

Figure S4 – hippocampal lipid mediators; Quantification of hippocampal eicosanoids. Two-way ANOVA for main diet and genotype effects was performed with a 95% confidence interval. P-values less than 0.05 are considered significant. Graphs are presented as mean ± standard deviation with all data points shown.

Figure S5 – microglial phagocytosis; (A-F) Phagocytosis of fluorescence labeled amyloid peptide (Aβ-488) by adult primary microglia from WT (A-C) and TG (D-F) mice was measured via flow cytometry. (A-B+D-E) Gating strategy for microglial cells. (C+F) Threshold setting for phagocytosing cells based on negative control without added Aβ-488 (not shown). (G) Representative hippocampal sections immunohistochemically stained with 4′,6-diamidino-2-phenylindole (DAPI) for cell nuclei (in blue), calcium-binding adapter molecule 1 (Iba1) for microglia (in red), cluster of differentiation 68 (CD68) (in green) and Thioflavin S for amyloid beta plaques (in magenta); scale size is 50 µm. (H) Total area of Iba1 immune reactive area per field of view. (I) Total area of CD68 immune reactive area per field of view. (J) CD68+ area normalized to Iba1+ area. (H-J) Two-way ANOVA for main diet and genotype effects was performed with a 95% confidence interval. P-values less than 0.05 are considered significant. Graphs are presented as mean ± standard deviation with all data points shown.

Figure S6 – Aβ-plaque pathology; (A) Representative hippocampal sections of TG mice immunohistochemically stained with 4′,6-diamidino-2-phenylindole (DAPI) for cell nuclei (in blue), Amytracker 520 (AmyT) for Aβ-plaques (in green) and lysosomal-associated membrane protein 1 (Lamp1) for dystrophic neurites (in white); scale size is 50 µm. Total plaque volume (B) and count (C) analysis performed with Imaris software. (D) Analysis of plaque size (area) distribution in the whole hippocampus, performed with ImageJ. Two-way ANOVA for main diet effects was performed with a 95% confidence interval, but was not significant. (E) Lamp1+ volume was measured as indicator for dystrophic neurites and normalized to plaque volume. (B,C,E) Student’s test with a 95% confidence interval was performed, without showing significant differences.
